# Prediction for Postpartum Hemorrhage of Placenta Previa Patients through MRI Using Self-Adaptive Edge Detection Algorithm

**DOI:** 10.1155/2021/8343002

**Published:** 2021-08-26

**Authors:** Yan Chen, Ting Xu

**Affiliations:** Department of Obstetrics, Changsha Hospital for Maternal & Child Health Care, Changsha City, 410007, Hunan Province, China

## Abstract

The study aimed to explore the application value of MRI images based on the optimized self-adaptive edge detection algorithm in the diagnosis of placenta previa and in the prediction of postpartum hemorrhage. Specifically, a self-adaptive edge detection algorithm was constructed based on optimized edge operators, with the nearest scale parameters analyzed. It was then used to process the MRI images of 36 patients with placenta previa. MRI images of different types of placenta previa were analyzed. The results found that the placenta of the complete placenta previa was attached to the lower wall of the uterus and covered the internal cervix in *U* shape, and the placenta adhered to the anterior and lower wall of the uterus, with widespread placenta accreta noted. With the results of cesarean section as the standard, it was observed that 2 cases of complete placenta previa were diagnosed as partial placenta previa. The diagnostic accuracy rate was 94.44%, which was not notably different from the results of cesarean section (*p* > 0.05). The postpartum hemorrhage rate and hysterectomy rate of complete placenta previa were higher than partial placenta previa and marginal placenta previa, and the difference was notable (*p* < 0.05), but no notable differences were noted in placenta adhesion, placenta accreta, neonatal death, and neonatal asphyxia between the three types of placenta previa (*p* > 0.05). The incidence of thinned myometrium, placenta penetrating the cervix, placenta accreta, and uneven placental signal in patients with postpartum hemorrhage was higher versus those without postpartum hemorrhage, and the difference was notable (*p* < 0.05). In a word, MRI images based on the self-adaptive edge detection algorithm can clearly show the status of placenta previa and exhibit better diagnosis effects and a higher accuracy rate. The thinned myometrium, the placenta penetrating the cervix, placenta accreta, and uneven placental signal may be the related risk factors for postpartum hemorrhage in patients with placenta previa.

## 1. Introduction

Placenta previa means that after 28 weeks of pregnancy, the placenta attaches to the lower part of the uterus, and even the lower edge of the placenta reaches or covers the internal cervix, and its position is lower than the fetal presentation site. This symptom is more common in multipara, especially grand multipara [1, 2]. According to the relationship between the placenta and the internal cervix, it can be divided into complete placenta previa, partial placenta previa, and marginal placenta previa [3]. The pathogenesis of placenta previa is not clear. Women who have had multiple miscarriages and curettage, puerperal infections, a history of cesarean sections, multiple pregnancies, poor habits (women who smoke or use drugs), or abnormal uterine morphology are at high risk [4]. It is clinically manifested as uninduced and painless recurrent vaginal bleeding during late pregnancy or near the time of labor. The amount of initial bleeding is generally not much; as the gestational age increases, bleeding can occur repeatedly, and the amount of bleeding will increase. In a few cases, fatal hemorrhages occur at the first time, leading to hemorrhagic shock [5]. Placenta previa is a common condition in the third trimester of pregnancy and, if not managed properly, can endanger the life of both mother and baby. Therefore, it is very important to predict placenta previa in advance.

In recent years, the rapidly developing MRI technology has been gradually applied to the diagnosis of placental diseases. Among them, MRI turbo spin echo (TSE) sequence and balanced fast field echo (BFFE) sequence are currently the best sequences to diagnose placenta previa. TSE can obtain better heavy T2 contrast, the image has high resolution for soft tissue, and the signal changes in the placenta are better displayed. However, it is difficult to distinguish the thickened and distorted low signal angiography in the placenta from the placental lobule interval and abnormal low signal bands [6–8]. BFFE sequence is a gradient echo sequence. Placenta MRI examination can better show the insertion of placental tissue into the muscular layer and localized ureterocele, with high accuracy. The tissue contrast of BFFE sequence images is the ratio of *T*2/*T*1. Due to the edge effect, the scanning speed is fast, and the interface between the placenta and the muscle layer can be well displayed. The thickened and twisted blood vessels in the placenta present high BFFE signal, but the soft tissue contrast of BFFE sequence is poor [9]. Conventional MRI scan includes sagittal, coronal, and transverse scans. The sagittal plane is the best observation plane for placenta accreta on the anterior wall of the uterus. The observation of placenta accreta on the lateral and posterior walls requires coronal and transverse scans [10]. Edge detection is a kind of computer vision processing technology for image processing and analysis. Many research reports have proposed related algorithms, including Prewitt, Kirsch, Robert, Canny, Gauss–Laplace, and self-adaptive edge detection. The self-adaptive edge detection algorithm can better locate the edge and suppress noise, but it requires a lot of computation, and the edge detection results of different images vary greatly [11, 12]. In the study, an edge operator based on morphological filtering was used to process and analyze MRI images of placenta previa patients.

In summary, MRI is widely used to diagnose placenta previa in clinical practice, but the quality of the original image is poor. Based on this, the optimized edge operator was used to construct a self-adaptive edge detection algorithm, which was used to process the MRI images of 36 patients with placenta previa. The image characteristics of different types of placenta previa were compared to comprehensively evaluate the application value of MRI images based on the optimized self-adaptive edge detection algorithm in the diagnosis of placenta previa and in the prediction of postpartum hemorrhage.

## 2. Materials and Methods

### 2.1. Research Subjects and the Grouping

36 patients with placenta previa who were treated in hospital from January 2020 to February 2021 were selected as the research subjects; they were aged 20–46 years old, with 25–39 gestational weeks, involving 11 cases of primipara and 25 cases of multipara. The study has been approved by the ethics committee of the hospital, and the patients and their families included in the study were informed and signed informed consent. The selection of samples complied with the diagnostic criteria and classification criteria of placenta previa in the Guidelines for Clinical Diagnosis and Treatment of Placenta Previa [13].

### 2.2. MRI Scan

MRI scans were performed before delivery (US GE 1.5T Signa HDi superconducting magnetic resonance, 8-channel phased array body coil). Before the examination, the patient was in a supine position with the bladder kept full. The scanning was from the lower edge of the pubic symphysis to the bilateral anterior superior iliac spine. Subsequently, the sagittal T1W1 scan and the sagittal and coronal plane T2W1 scans were performed. T1W1 scanning parameters were TE15 ms, TR800 ms, matrix 256 × 256, and layer thickness 2.5 mm. The scanning parameters of T2W1 were TE 1.45 ms, TR 3.5 ms, and layer thickness 6 mm.

### 2.3. The Self-Adaptive Edge Detection Algorithm Based on Optimized Edge Operators

Edge fractures often occur when the traditional edge detection algorithm extracts edge features of MRI images, leading to poor quality of MRI images to display human body structures and soft tissue. In the study, an edge operator with antinoise filtering characteristics is used. Supposing that there is an image *g*(*i*, *j*) and the structural element is *J*, then the following equation is obtained according to the basic operation theorem of morphology.

(1) $$\begin{array}{c} g⊕J\geq g*J\geq g\geq g\circ J\geq g\Theta J. \end{array}$$

Therefore, the traditional morphological gradient edge detection operator can be expressed as follows.

(2) $$\begin{array}{c} \left\{\begin{array}{c} p1=g⊕J-g,\\ p2=g-g\Theta J,\\ p3=g⊕J-g\Theta J,\\ p4=g-g\circ J,\\ p5=g*J-g,\\ p6=g*J-g\circ J. \end{array}\right) \end{array}$$

To endow the traditional morphological gradient edge detection operator with antinoise filtering characteristics, the above operators are combined in cascade to obtain the following equation.

(3) $$\begin{array}{c} \left\{\begin{array}{c} q1=\left(g*J\right)\circ J-\left(g*J\right)\Theta J,\\ q2=\left(g\circ J\right)⊕J-\left(g\circ J\right)*J. \end{array}\right) \end{array}$$

**Figure 1:**
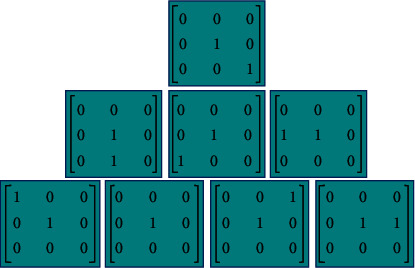
Schematic diagram of structural elements.

The above operators are instrumental in processing bright and dark noises, but the edges obtained are relatively blurry. Hence, further improvement is needed.

(4) $$\begin{array}{c} q_{\max}=\max\left\{q1,q2\right\},\\ q_{\min}=\min\left\{q1,q2\right\},\\ Q=\kappa *q_{\max}+\lambda *q_{\min}, \end{array}$$

where *q*_max_ represents the suppression of the operator on dark noise and *q*_min_ represents the suppression effect of the operator on bright noise. *κ* and *λ* are the adjustment parameters, and *κ*+*λ*=1. Subsequently, the structural element is designed (Figure 1). The structural element has a scale of 3 and includes 8 directions.

Assuming that the image size is 3 × 3, it can be expressed as $\left\{\begin{array}{ccc} d8 & d1 & d2\\ d7 & d0 & d3\\ d6 & d5 & d4 \end{array}\right\}$, where *d*0 is the gray value of the central pixel, and *d*1, *d*2,…, *d*8 represent the gray values of the neighboring pixels. The difference squares of the image neighborhood are accumulated to obtain the overall gray-scale difference characteristics of the image in different directions, with the weights of the structural elements in different directions calculated. The equation is as follows.

(5) $$\begin{array}{c} h_{m}=\sum_{i=1}^{R-1}\sum_{j=2}^{S-1}g_{Qm}\left(i,j\right),\;m=1,2,3,4, \end{array}$$

(6) $$\begin{array}{c} v_{Qm}=\frac{h_{m}}{\sum_{m=1}^{4}h_{m}},\;m=1,2,3,4. \end{array}$$

To further extract the edge details of the image, the concept of gray mutation direction is introduced, and the center pixel is calculated as follows.

The square of the difference with the neighboring pixels is calculated to get the gray-level difference characteristics in each direction, and then, the following equation is obtained.

(7) $$\begin{array}{c} l_{x}=\sum_{i=2}^{R-1}\sum_{j=2}^{S-1}l_{Qx}\left(i,j\right), \end{array}$$

where *l* represents the characteristics of gray-scale difference, *x*=1,2,3,4,5,6,7,8. Based on equations (6) and (7), the corresponding structural element weight calculation method is as follows.

(8) $$\begin{array}{c} v_{1}=\frac{l_{5}}{l_{5}+l_{6}}*v_{Q3},\\ v_{2}=\frac{l_{7}}{l_{7}+l_{8}}*v_{Q4},\\ v_{3}=\frac{l_{1}}{l_{1}+l_{2}}*v_{Q1},\\ v_{4}=\frac{l_{3}}{l_{3}+l_{3}}*v_{Q2},\\ v_{5}=v_{Q3}-v_{1},\\ v_{6}=v_{Q4}-v_{2},\\ v_{7}=v_{Q1}-v_{3},\\ v_{8}=v_{Q2}-v_{4}. \end{array}$$

**Figure 2:**
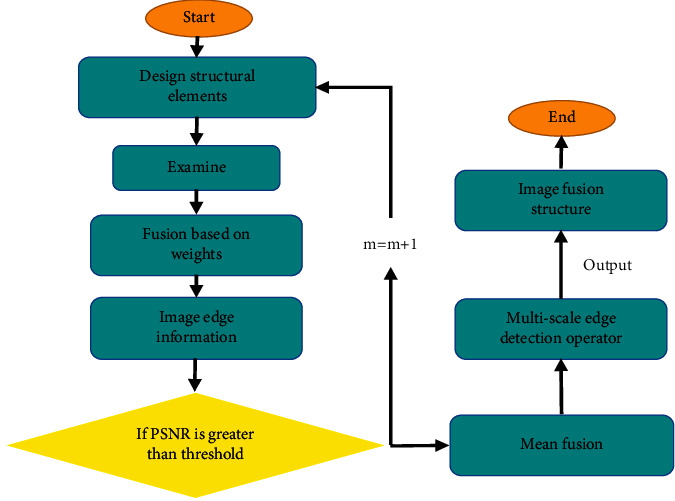
Algorithm flowchart.

The above are the weights of structural elements in 8 directions, and each weight is applied to the constructed edge operator to obtain edge information of MRI images at different scales. The mean of edge information is then fused, and the final fusion result of the MRI image is as follows.

(9) $$\begin{array}{c} \text{Mu}Q\left(i,j\right)=\sum_{x=1}^{S-1}\frac{Q_{x}\left(i,j\right)}{n}, \end{array}$$

where Mu*Q* represents the multiscale edge detection operator. The specific algorithm flow is shown in Figure 2 . First, eight-direction structural elements with a scale of (2*m*+1)*∗*(2*m*+1), *m*=1,2,3,…, *n* are designed first, which are then detected. The detection results are fused based on the weights to obtain the edge information of the image. If the peak signal-to-noise ratio (PSNR) value is greater than the set threshold, subsequent calculations are performed. Otherwise, let *m* = *m* + 1, go back to the first step. The mean of edge is then fused to obtain the image fusion result.

### 2.4. Simulation Experiment

Experimental environment: processor 2.8 GHz, the computer memory is 1 GB, and the development environment is Matlab7.2.

In order to analyze the edge detection effects of structural elements of different scales, the scales are set to 3*∗*3, 4*∗*4, 5*∗*5, and 6*∗*6, respectively, and the constructed algorithm is used for MRI image processing.

The indicators used to evaluate the image quality include mean square error (MSE), structural similarity index (SSIM), PSNR, and running time. The equation is as follows.

(10) $$\begin{array}{c} \text{MSE}\left(E,R\right)=\frac{\sum_{i=1}^{P_{1}}\sum_{j=1}^{P_{2}}{\left(R\left(i,j\right)-E\left(i,j\right)\right)}^{2}}{P_{1}\times P_{2}},\\ \text{PSNR}\left(E,R\right)=10\mathrm{lg}\frac{256\times 256}{\text{MSE}},\\ \text{SSIM}\left(E,R\right)=\frac{2\alpha_{1}\alpha_{2}+C_{1}}{\alpha_{1}^{2}+\alpha_{2}^{2}+C_{1}}\frac{2\beta_{12}+C_{2}}{\beta_{1}^{2}+\beta_{2}^{2}+C_{2}}, \end{array}$$

where *R* represents the original image, and *E* represents the processed image. The image size is *P*_1_ × *P*_2_, *C* is a constant, *β*_12_ is the covariance, and *α* is the image mean.

### 2.5. Observation Indicators

The algorithms with structural elements of different scales are recorded for MSE, SSIM, PSNR, and running time. Placental MRI images were analyzed by two MRI diagnosticians with 5 years of experience using a double-blind method. The patient's MRI was observed for thinned the myometrium, the placenta penetrating the cervix, placenta accreta, uneven placental signal, and uterine confined carina. The placenta previa patients were classified (complete placenta previa, partial placenta previa, and borderline placenta previa). The placenta previa of different types were compared for the postpartum hemorrhage and pregnancy outcome (hysterectomy, placenta adhesion, placental implantation, neonatal death, and neonatal asphyxia).

### 2.6. Statistical Methods

The experimental data were processed using SPSS19.0 statistical software, the measurement data were expressed as the mean ± standard deviation $\left(\bar{x}\pm s\right)$, the count data were expressed as a percentage (%), and the pairwise comparison of indicators was performed by analysis of variance. (*p* < 0.05) was the threshold for significance.

## 3. Results

### 3.1. The Quality of MRI Images under Parameters of Different Scales

**Figure 3:**
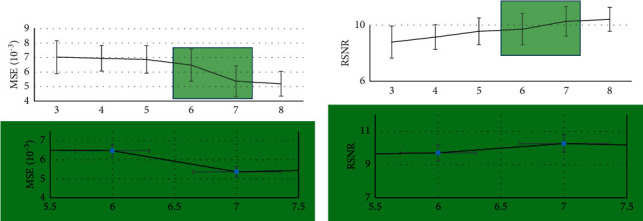
MSE and PSNR of the algorithm at different scales. (a) MSE. (b) PSNR.

**Figure 4:**
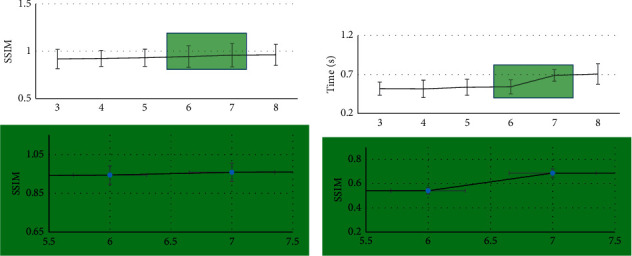
SSIM and running time of the algorithm at different scales. (a) SSIM. (b) Running time.

As shown in Figures 3 and 4, when the scale of the structural element increased, the MSE of the MRI image after processed by the algorithm decreased, and it decreased by a wide margin when the scale was between 6 and 7. When the scale of the structural element increased, the PSNR and SSIM of the MRI image after processed by the algorithm increased accordingly, and the overall trend was relatively stable. When the scale of structural element increased, the running time of the algorithm was prolonged. The running time increased by a small margin at 3–6 o'clock and by a wide margin at 7 o'clock. Taken together, in actual situation, the corresponding thresholds need to be set according to experimental requirements.

### 3.2. MRI Image Analysis of Different Types of Placenta Previa

**Figure 5:**
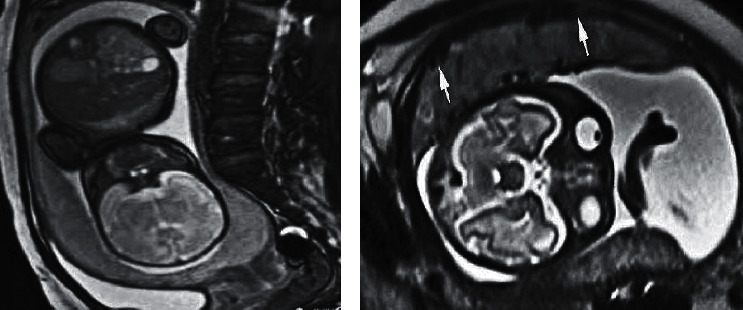
The MRI image of a complete placenta previa case. (a) The sagittal plane. (b) The transverse plane.

Figure 5 is the MRI image of a case with complete placenta previa (female, 40 years old, 32-week gestation age, 4th pregnancy with one child). The placenta was attached to the lower wall of the uterus in the sagittal plane, covering the internal cervical in *U* shape. The transverse scan showed that the placenta adhered to the anterior inferior wall of the uterus, and widespread placenta accreta was noted, consistent with postoperative pathology.

**Figure 6:**
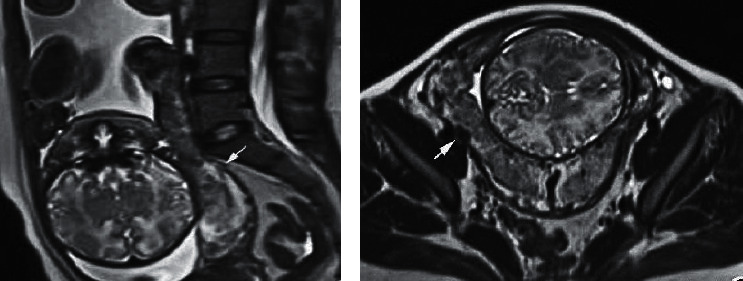
The MRI image of a partial placenta previa case. (a) The sagittal plane. (b) The transverse plane.

Figure 6 is the MRI image of a partial placenta previa case (female, 25 years old, 35-week gestational age, 2nd pregnancy no child). The sagittal scan showed that the uterine conjunctiva disappeared, the basal layer of the uterus was thinned, the placental signal was uneven, and placental villi protruded into the myometrium (arrow), showing partial placenta previa combined with multisegmental placenta accreta. The transverse scan showed that the placenta accreta occurred on the right posterior lower wall of the uterus (arrow), which did not penetrate the serosal layer, and there were multiple flowing void signals in the placenta, consistent with the findings during the operation.

**Figure 7:**
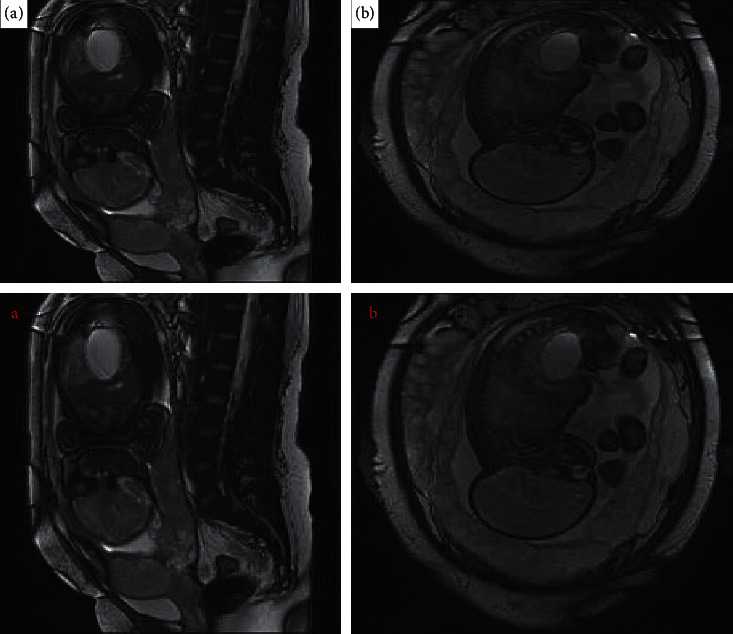
The MRI image of a marginal placenta previa case. (a) The sagittal plane. (b) The transverse plane.

Figure 7 is the MRI image of a marginal placenta previa case (female, 31 years old, 29-week gestational age, 1st pregnancy no child). The sagittal scan showed the thinned myometrium and unclear three-layer structure. The junction zone with slightly low signals was blurry, and the edge of the placenta was close to the internal cervix, but not entering the internal cervix. The horizontal axis plane showed that the placenta locally broke through the uterine serosal layer, the placenta was heterogeneous, with cord-like low signal shadow and multiple flowing void shadows noted.

### 3.3. Diagnosis Results of MRI Images

**Figure 8:**
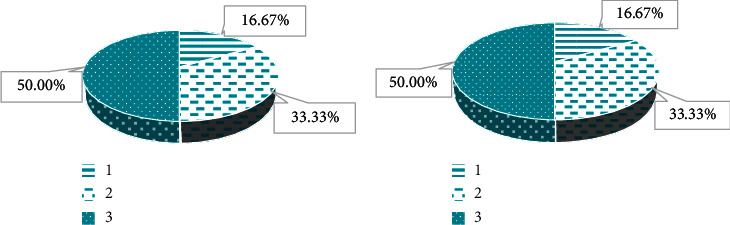
The diagnosis results of cesarean section and MRI images. 1 represents the complete placenta previa; 2 represents the partial placenta previa; 3 represents the marginal placenta previa.

With the results of cesarean section as the standard (Figure 8(a)), among 36 patients, there were 19 cases of complete placenta previa, 11 cases of partial placenta previa, and 6 cases of marginal placenta previa. The MRI diagnosis results (Figure 8(b)) showed that there were 17 cases of complete placenta previa, 13 cases of partial placenta previa, and 6 cases of marginal placenta previa. Obviously, 2 cases of complete placenta previa were diagnosed as partial placenta previa. The diagnostic accuracy rate was 94.44%, which was not notably different from the results of cesarean section (*p* > 0.05).

### 3.4. The Postpartum Hemorrhage and Pregnancy Outcome

**Figure 9:**
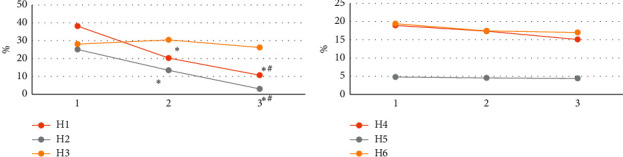
The postpartum hemorrhage and pregnancy outcome of different types of placenta previa. 1 represents the complete placenta previa; 2 represents the partial placenta previa; 3 represents the marginal placenta previa; H1, postpartum hemorrhage; H2, hysterectomy; H3, placental adhesions; H4, placenta accreta; H5, neonatal death; H6, neonatal asphyxia.  ^*∗*^Notable difference compared to the complete placenta previa (*p* < 0.05). ^*#*^Notable difference compared to the partial placenta previa (*p* < 0.05).

As shown in Figure 9, the incidence of postpartum hemorrhage and hysterectomy of complete placenta previa was higher than that of partial placenta previa and marginal placenta previa, and the difference was notable (*p* < 0.05). The incidence of postpartum hemorrhage and hysterectomy of partial placenta previa was higher than that of marginal placenta previa, and the difference was notable (*p* < 0.05). No notable differences were noted in placenta adhesion, placenta accreta, neonatal death, and neonatal asphyxia between the three types of placenta previa (*p* > 0.05).

### 3.5. Comparison of MRI Characteristics between Patients with Postpartum Hemorrhage and Patients without Postpartum Hemorrhage

**Figure 10:**
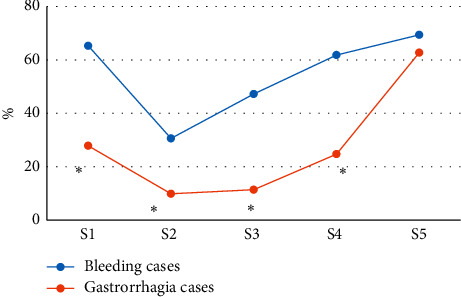
Comparison of MRI characteristics between patients with postpartum hemorrhage and patients without postpartum hemorrhage. S1, thinned myometrium; S2, placenta penetrating the cervix; S3, placenta accreta; S4, uneven placental signal; S5, uterine confined carina.  ^*∗*^Notable differences compared with cases of postpartum hemorrhage (*p* < 0.05).

As shown in Figure 10, according to whether the patient had postpartum hemorrhage, the incidence of thinned myometrium, placenta penetrating the cervix, placenta accreta, and uneven placental signal was higher in patients with postpartum hemorrhage versus those without postpartum hemorrhage, and the difference was notable (*p* < 0.05). No notable difference was noted in the incidence of uterine confined carina between patients with postpartum hemorrhage versus those without postpartum hemorrhage (*p* > 0.05).

## 4. Discussion

Placenta previa is the main clinical cause of postpartum hemorrhage in pregnant women, which can lead to hemorrhagic shock and even death. Therefore, early diagnosis and intervention of placenta previa is of great significance for the mother and fetus. The clinical diagnosis of placenta previa often relies on clinical manifestations and ultrasound imaging. Although ultrasound is good for the diagnosis of placenta previa in pregnant women, it is easily affected by objective conditions such as overfilling of the bladder and fetal position [14]. In recent years, MRI has performed extremely well in obstetrics. It can clearly show the placenta and cervix and improve the accuracy of diagnosis. In the study, a self-adaptive edge detection algorithm was constructed based on the optimized edge operator to process the MRI images, with the nearest scale parameters analyzed. The results showed that when the scale of the structural element increased, the MSE of the MRI image decreased, and the MSE decreased obviously between 6 and 7, while the PSNR and SSIM increased accordingly, and the overall trend was relatively stable. Furthermore, when the scale of structural elements increased, the running time of the algorithm also prolonged. The running time increased by a small margin at 3–6 o'clock and increased by a wide margin between 6 and 7 o'clock, which showed that although the image quality was improved by increasing the scale of structural elements, it also prolonged the running time required for image processing. Hence, a threshold needed to be set according to the actual situation [15].

The self-adaptive edge detection algorithm was used to process MRI images of 36 patients with placenta previa. It was found that the image characteristics of different types of placenta previa were apparently distinct. The placenta of complete placenta previa was attached to the lower wall of the uterus and covered the cervix in *U* shape. The placenta adhered to the anterior inferior wall of the uterus, and widespread placenta accreta was noted. For partial placenta previa, the uterine conjunctive zone disappeared, the basal layer of the uterus was thinned, and the placental signal was uneven. The placental villi that protruded into the myometrium, multisegment placenta accreta, and flowing void were noted. Marginal placenta previa showed the limited thinning of the myometrium and unclear three-layer structure. The edge of the placenta was close to the internal cervix, but did not enter the internal cervix. The placenta was heterogeneous, and cord-like low signal shadow and multiple flowing void shadow were noted [16]. With the results of cesarean section as the standard, it was observed that 2 cases of complete placenta previa were diagnosed as partial placenta previa. The diagnostic accuracy rate was 94.44%, which was not notably different from the results of cesarean section (*p* > 0.05). It was in line with the results of Chen et al. [17], indicating that MRI images based on the self-adaptive edge detection algorithm had good diagnostic effects for placenta previa in pregnant women, and the accuracy rate was high. The postpartum hemorrhage rate and hysterectomy rate of complete placenta previa were higher than partial placenta previa and marginal placenta previa, and the difference was notable (*p* < 0.05), but no notable differences were noted in placenta adhesion, placenta accreta, neonatal death, and neonatal asphyxia between the three types (*p* > 0.05). This was different from the research results of Su and Chen [18]. It may be due to the lower position of the complete placenta previa, and the cervix would be completely covered by the placenta tissue. The internal cervix has less muscle tissue and poor contraction ability. As a result, it is not easy to stop bleeding, and it is necessary to remove the uterus [19]. The incidence of thinned myometrium, placenta previa, placenta accreta, and uneven placental signal was higher in patients with postpartum hemorrhage versus those without postpartum hemorrhage, and the difference was notable (*p* < 0.05), which suggested that the thinned myometrium, the placenta penetrating the cervix, placenta accreta, and uneven placental signal may be the related risk factors for postpartum hemorrhage in patients with placenta previa, which can be used to predict the occurrence of postpartum hemorrhage in patients.

## 5. Conclusion

In the study, a self-adaptive edge detection algorithm was constructed based on the optimized edge operator, with the nearest scale parameters analyzed. It was then used to optimize MRI images of 36 patients with placenta previa. It was found that the MRI image based on the self-adaptive edge detection algorithm can clearly show the status of placenta previa, exhibiting better diagnosis effects and higher accuracy rate. The thinned myometrium, the placenta penetrating the cervix, placenta accreta, and uneven placental signal may be the related risk factors for postpartum hemorrhage in patients with placenta previa. However, this study does not further analyze the influencing factors of postpartum hemorrhage in patients with placenta previa, and the included factors are all imaging features, and clinical data are not included. Therefore, in the follow-up, basic data of more patients need to be involved to deeply explore the influencing factors of postpartum hemorrhage. All in all, the results of this study provide a scientific theoretical support for the clinical diagnosis of placenta previa and the prevention of postpartum hemorrhage.

## Data Availability

The data used to support the findings of this study are available from the corresponding author upon request.
